# Changes in the distribution pattern of Claudin tight junction proteins during the progression of mouse skin tumorigenesis

**DOI:** 10.1186/1471-2407-7-196

**Published:** 2007-10-18

**Authors:** Azadeh Arabzadeh, Tammy-Claire Troy, Kursad Turksen

**Affiliations:** 1 Ottawa Health Research Institute, Ottawa, Ontario, K1Y 4E9 Canada; 2 Department of Cellular and Molecular Medicine, Faculty of Medicine, University of Ottawa, Ottawa, Ontario, K1H 8M5 Canada; 3 Department of Medicine, Divisions of Dermatology and Endocrinology, Faculty of Medicine, University of Ottawa, Ottawa, Ontario, K1H 8M5 Canada; 4 Department of Obstetrics, Gynaecology, Division of Reproductive Endocrinology, Faculty of Medicine, University of Ottawa, Ottawa, Ontario, K1H 8M5 Canada

## Abstract

**Background:**

Despite the fact that morphological and physiological observations suggest that the tight junction (TJ)-based permeability barrier is modified/disrupted in tumorigenesis, the role of members of the Claudin (Cldn) family of TJ proteins is not well-understood. Using a well-established two-stage chemical carcinogenesis model, we investigated the temporal and spatial changes in expression of those Cldns that we have previously demonstrated to be important in epidermal differentiation and the formation of the epidermal permeability barrier, i.e., Cldn1, Cldn6, Cldn11, Cldn12 and Cldn18.

**Methods:**

The lower dorsal backskin of mice was treated topically with 7,12-dimethylbenz(*a*)anthracene (DMBA; 0.25 mg/ml in acetone) and following a 10-day incubation period, 12-O-tetradecanoyl-phorbol-13-acetate (TPA; 25 μg/ml in acetone) was applied three times a week to the same area. Backskin samples were dissected 2, 4, 6, 8 and 12 weeks after the initiation of the experimental protocol and immunohistochemistry was performed on sections using antibodies against the following: Cldn1, Cldn6, Cldn11, Cldn12, Cldn18, Ki67 and CD3.

**Results:**

Our data indicate that along with the changes in epidermal cell morphology and differentiation that occur during tumor formation, there is a dramatic change in Cldn distribution consistent with cell polarity and barrier selectivity changes. Specifically, in the early stages of DMBA/TPA treatment, the suprabasal-specific Cldns occupy an expanded zone of expression corresponding to an increased number of suprabasal epidermal cell layers. As tumorigenesis progressed, the number of suprabasal epidermal layers positive for Cldn6, Cldn11, Cldn12 and Cldn18 was reduced, especially in the lower strata of the expanded suprabasal zone. In addition, a variably reduced cell membrane association of those differentiation-specific Cldns was observed, especially within the infiltrating epidermal structures. In contrast, Cldn1 (which is normally expressed in all the living layers of the epidermis) remained restricted to the cell membrane throughout the tumorigenesis protocol. However commencing 2 weeks after treatment there was a marked decrease in the number of Cldn1-positive basal cells, and the zone of Cldn1-null epidermal cells was expanded up into the lower stratified epidermis throughout the progression of DMBA/TPA treatment. In addition, there was no Cldn1 localization in the infiltrating epidermal structures of the tumorigenic epidermis.

**Conclusion:**

This is the first demonstration of the changes in Cldn expression in the progression of DMBA/TPA-induced skin tumors; however further investigation into the molecular mechanisms regulating the observed changes in barrier selectivity during tumorigenesis is required.

## Background

Disruption of epithelial cell polarity and cell-cell junctions with concomitant changes in the expression of junctional proteins during primary tumor formation is considered to be a hallmark of cancer cell invasion and metastasis [1]. Amongst the junctional complexes, the role in tumor formation of specific tight junction (TJ) proteins essential for cell polarity and the formation and maintenance of heterogeneous permeability barriers is not well understood. In vivo studies have demonstrated that the epidermis is characterized by a defined differentiation-dependent expression of Claudins (Cldns), a family of tetraspan membrane proteins that comprise a major component of TJ fibrils essential to the structure and function of TJs [2-6]. Recent studies have also shown that changes in the distribution pattern of diverse Cldns may contribute to changes observed in cell permeability [7-10]. However, no systematic analysis of the expression and/or localization of various Cldns during skin tumorigenesis is yet available.

Understanding the molecular basis of skin tumor development has been greatly facilitated by the use of animal model systems in which tumor development can be carefully controlled [11]. For example, the classical mouse two-stage carcinogenesis model provides an excellent system in which to study the stages and molecular mechanisms involved in squamous cell carcinoma [12]. The chemical carcinogenesis process can be divided into three distinct phases: initiation, promotion and progression. Initiation results from exposure to a mutagenic carcinogen, followed by the application of a promoter to alter gene expression and increase cell proliferation to ultimately result in tumor formation. Therefore, we used this model to elucidate the changes occurring in Cldn expression during the progression of epithelial tumors.

## Methods

### DMBA/TPA treatment

The coat on the dorsal side of one-month-old CD1 wild type mice was shaved one day prior to the initiation of the experimental protocol and mice were shaved once a week as required until the coat failed to re-grow. The lower dorsal backskin of mice was treated topically with 7,12-dimethylbenz(*a*)anthracene (DMBA; 0.25 mg/ml in acetone) and following a 10-day incubation period, 12-O-tetradecanoyl-phorbol-13-acetate (TPA; 25 μg/ml in acetone) was applied three times a week to the same area. Experimental results were highly reproducible in three independent assays, each comprising three mice per time point (treated vs. control) and a minimum of 2–3 biopsies per mouse. All animal studies were conducted according to the regulations of the Canadian Council on Animal Care.

### Sample Collection

Backskin samples (~1 cm^2^) were dissected from the lesion and/or tumor regions of the mid-dorsal backskin from DMBA/TPA-treated mice as well as their vehicle-treated controls; sampling was done 2, 4, 6, 8 and 12 weeks after the initiation of the experimental protocol. Since the fixation method routinely used in our laboratory is rather stringent (see below) and not appropriate for all antibodies, frozen sections were required for Cldn1, Ki67 and CD3 immunostaining [2]. All other immunolocalization and histology (Hematoxylin & Eosin; H&E) were performed on paraffin sections. For frozen sections: skin samples were embedded in HistoPrep™ and solidified in dry ice-chilled isopentane. Sections (5 μm) on slides were warmed at room temperature for 3 minutes, and then fixed for 10 minutes in methanol at -20°C, followed by washing in PBS prior to immunostaining. For paraffin sections: skin samples were fixed for 12–16 hours in Bouin's fixative (75% saturated picric acid, 20% formaldehyde and 5% glacial acetic acid) at room temperature, followed by ethanol dehydration (30%, 50%, 70%, 95%, 100%), paraffin embedding and sectioning (5 μm). Prior to H&E and immunostaining, sections were dewaxed and rehydrated followed by antigen unmasking and washing steps [5].

### Immunohistochemistry

Paraffin and frozen sections were blocked for non-specific antibody binding by a 30-minute room temperature incubation (10% goat serum, 0.8% BSA, 1% gelatin in PBS) followed by several washes in wash buffer (0.8% BSA, 1% gelatin in PBS). Primary antibodies appropriately diluted in incubation buffer (1% goat serum, 0.8% BSA, 1% gelatin in PBS) were applied for 1–2 hours at room temperature; antibodies against the following were used: Ki67 (1:25) (cat. # ab833; abcam, Cambridge, MA), CD3 (1:100) (cat. # 555273; BD Biosciences, Franklin Lakes, NJ), Cldn6 (1:50) (custom antibody generated from hen #3677 against mouse Cldn6 sequence-CYSTSVPHSRGPSEYPTKNYV, Aves Labs, Inc., San Diego, CA), Cldn11 (1:50) (custom antibody generated from hen #3680 against mouse Cldn11 sequence-CRKMDELGSK, Aves Labs, Inc.), Cldn12 (1:50) (custom antibody generated from hen #5186 against mouse Cldn12 sequence-CZRKLRLITFNRNEKNLTIYT, Aves Labs, Inc.), Cldn18 (1:50) (custom antibody generated from rabbit #A9953 against mouse Cldn18 sequence-CRTEDDEQSHPTKYDYV, Open Biosystems, Huntsville AL) and Cldn1 (6:100) (cat. #71–7800; Invitrogen, Burlington, Canada). Following incubation in wash buffer, secondary antibodies against rabbit, rat and chicken conjugated to FITC (Jackson ImmunoResearch Laboratories, West Grove, PA) were used at a 1:50 dilution in incubation buffer for 1 hour at room temperature. Images were acquired using a Zeiss Axioplan 2 brightfield/fluorescence capable microscope outfitted with an AxioCam camera and Axio Vision 2.05 software (Carl Zeiss Canada Ltd, Toronto, Canada) before processing with Adobe Photoshop version 7.0 (Adobe Systems, Inc., San Jose, CA).

## Results and discussion

### Characteristics of skin tumor progression

**Figure 1:**
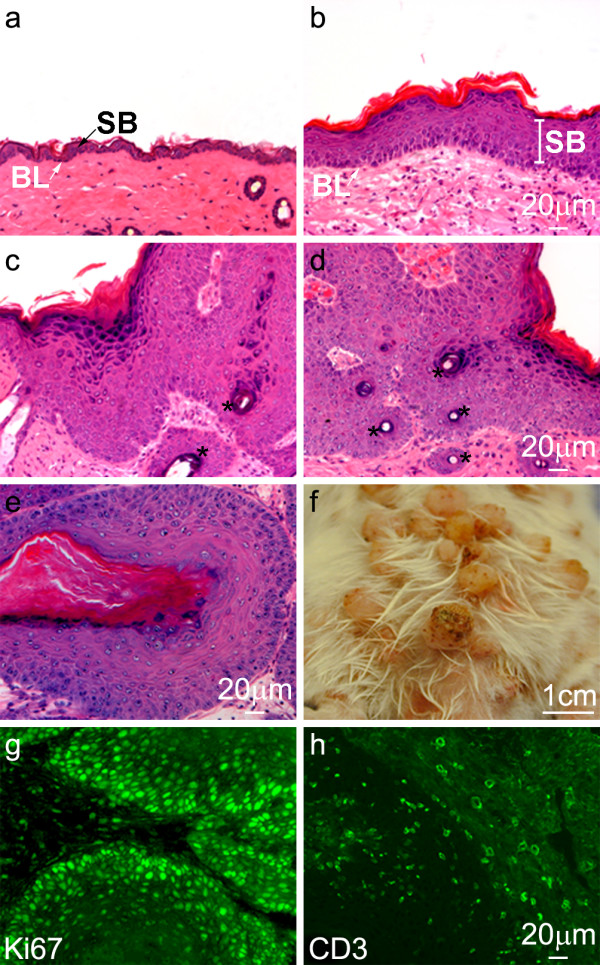
**Histological characteristics of skin tumor formation**. H&E staining of the vehicle-treated epidermis after 12 weeks is shown (**a**). The epidermis after 4 (**b**), 6 (**c**), 8 (**d**) and 12 (**e**) weeks of the classical two-stage chemical carcinogenesis protocol revealed striking abnormalities in epidermal architecture consistent with tumor formation, including the appearance of pseudohorn cysts (marked with stars). The dorsal skin of the mouse after 12 weeks of DMBA/TPA treatment is shown (**f**). Both Ki67-positive cells (**g**) and CD3-positive infiltrates (**h**) were evident throughout the experimental protocol (12 weeks is shown). BL: basal layer; SB: suprabasal compartment.

When treated with vehicle (acetone) only for 12 weeks, the epidermis (Figure 1a) was morphologically reminiscent of the normal, untreated epidermis in age-matched samples (not shown). Initiation with DMBA followed by 2 to 12 weeks of TPA treatment caused the epidermis progressively to display abnormalities in morphological architecture consistent with tumor formation, including a much thicker suprabasal compartment, an expanded stratum corneum and a more dispersed granular layer (Figure 1b–e). In addition, characteristic invaginations of epidermal structures into the dermis were evident (Figure 1c–e), as were pseudohorn cysts (Figure 1c–d, marked with stars); a photograph depicting the dorsal side of the DMBA/TPA-treated mouse after 12 weeks is shown (Figure 1f). Throughout the experimental protocol, immunofluorescence confirmed the expression of Ki67 (Figure 1g), a nuclear protein expressed by proliferating cells during all phases of the cell cycle, and the T-cell receptor-associated CD3 complex (Figure 1h), indicative of the proliferative and immune infiltrative states of the treated epidermis. The expression and localization of keratins and epidermal terminal differentiation markers were also progressively disrupted as has been previously described (not shown) [13].

### Expression of the suprabasal Cldns in skin tumorigenesis

**Figure 2:**
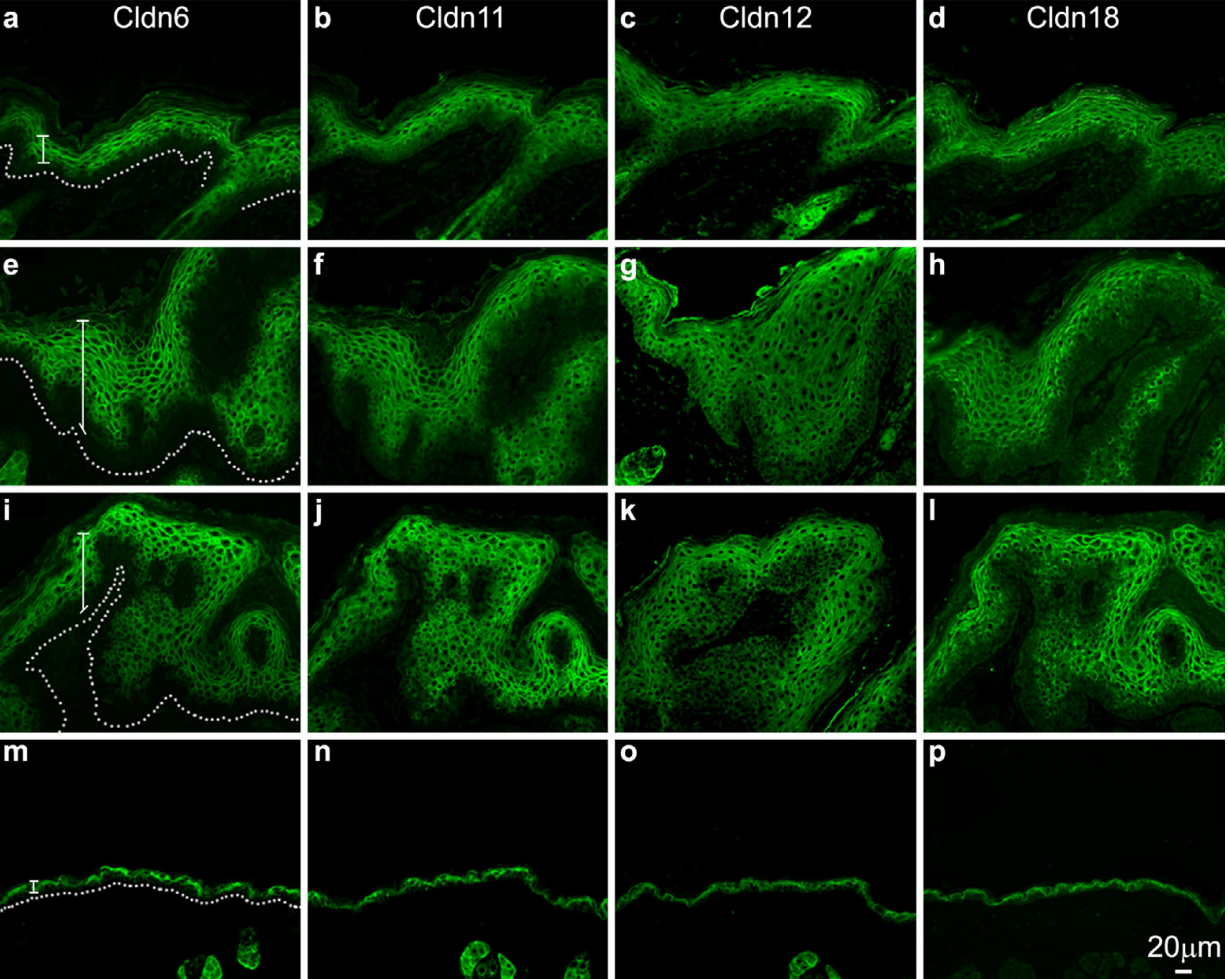
**Suprabasal-specific Cldns in epidermal tumor progression**. Cldn6, Cldn11, Cldn12 and Cldn18 expression changes during epidermal tumorigenesis to occupy a gradually expanding zone of expression as observed after 2 (**a-d**), 6 (**e-h**) and 12 (**i-l**) weeks of the carcinogenesis protocol. While the localization of these suprabasal-specific Cldns shifted to varying degrees away from the cell membrane, there was a correspondingly varied reduction in the number of Cldn-positive epidermal cell layers in the lower strata of the epidermis after 6 and 12 weeks. The acetone-treated epidermis (after 12 weeks) is shown (m-p). A dotted line marks the base of the epidermal basal layer and the suprabasal zone is indicated with a bracket.

**Figure 3:**
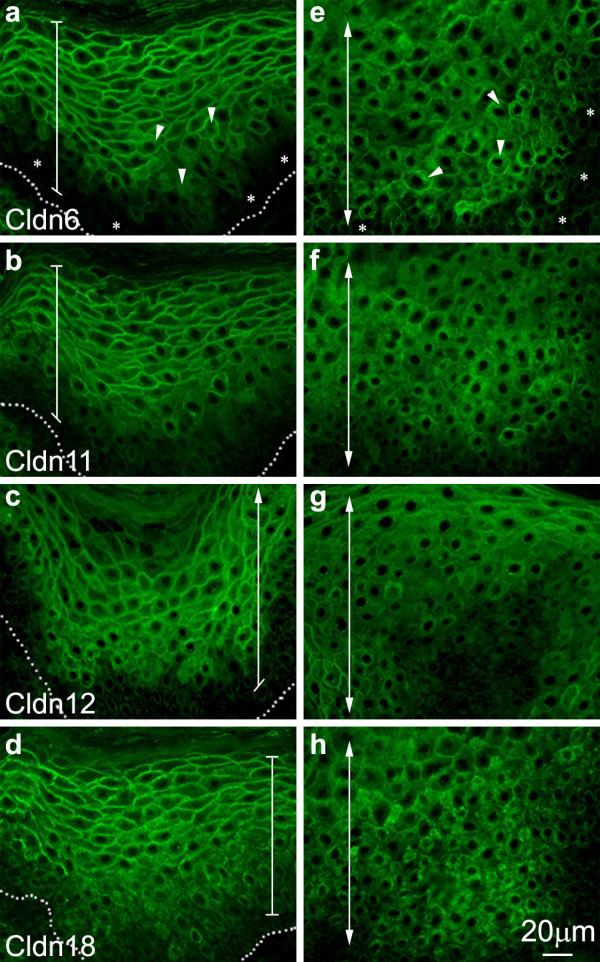
**Differential Cldn localization in the tumorigenic epidermis**. Higher magnification immunolocalization images of the epidermis (**a-d**) as compared to the characteristic epidermal structures infiltrating the dermis (**e-h**) better demonstrate the differences in Cldn localization 8 weeks after the initiation of the DMBA/TPA protocol. Membranous Cldn association was more prominent for each Cldn, albeit to varying degrees, in the upper strata of the epidermis; whereas in the lower strata and the epidermal invaginations Cldn localization was less membranous in nature and Cldn-null epidermal cells were more frequently observed. A dotted line marks the basal layer, and a bracket encompasses the epidermal suprabasal compartment in a-d. A double-ended arrow marks the suprabasal compartment in e-h to demonstrate that the entire panel represents suprabasal cells; arrowheads point to representative areas of non-membranous Cldn localization, and stars designate areas of Cldn-negative epidermal cells.

We next analyzed the expression of those Cldns we have previously demonstrated to be important in epidermal differentiation and the formation of the epidermal permeability barrier [2,4,5,14]. The distribution of Cldn6, Cldn11, Cldn12 and Cldn18 was indistinguishable in the normal (not shown) and vehicle-treated (Figure 2m–p) epidermis after 12 weeks, occupying the entire suprabasal compartment (in Figure 2 the basal layer is marked by a dotted line and the suprabasal compartment is indicated with a bracket). After 2 weeks of DMBA/TPA treatment (Figure 2a–d), on the other hand, the suprabasal compartment was expanded, with a corresponding expanded zone of Cldn expression in all the suprabasal layers. However, after 6 (Figure 2e–h), 8 (Figure 3a–d) and 12 (Figure 2i–l) weeks, the treated epidermis displayed a marked reduction in the number of suprabasal cell layers staining positively for Cldn6, Cldn11, Cldn12 and Cldn18; the loss of staining was evident in the lower suprabasal zone (Figure 3; representative areas of Cldn-negative suprabasal cells are marked with stars). In addition there was an obvious but somewhat variable shift in the subcellular localization of all four of these Cldns away from the cell membrane that was especially evident in the lower suprabasal layers of the DMBA/TPA-treated epidermis after 8 weeks (Figure 3a–d, arrowheads illustrate representative areas where Cldn association is not restricted to cell membranes). Although all of the suprabasal-specific Cldns assayed showed some degree of non-membranous labeling after 8 weeks of treatment, this was especially true for Cldn12 and Cldn18 where the subcellular shift extended higher into the suprabasal zone than for Cldn6 and Cldn11.

In the characteristic epidermal structures infiltrating the dermis of tumors after 8 weeks of treatment, the subcellular translocalization of the suprabasal Cldns away from the cell membrane was even more apparent, with a nearly complete loss of membrane labeling (Figure 3e–h; the suprabasal compartment is marked with a double-ended arrow to demonstrate that the view encompasses only suprabasal cells and arrowheads point to representative areas of non-membranous Cldn labeling). In addition, there were many Cldn6-, Cldn11-, Cldn12- and Cldn18-negative suprabasal cell layers evident (note that in Figure 3, the stars highlight areas of Cldn-negative epidermal cells).

The expanded suprabasal Cldn expression compartment in early DMBA/TPA treatment, followed by the loss of Cldn6, Cldn11, Cldn12 and Cldn18 in the lower strata of the suprabasal compartment as well as the reduced cell membrane association of those Cldns primarily associated with the stratifying/differentiating layers of the normal epidermis raises the question of whether such changes are obligatory for the promotional stage of skin tumor formation. The change in Cldn expression and localization may also impart new permeability properties to the affected and surrounding epidermal cells resulting in their hyperplastic conversion. Our results are consistent with the hypothesis of Daugherty et al. [15] that undifferentiated cells with poor barrier function exhibit a significant intracellular Cldn pool. In addition to transcriptional and translational regulation of expression levels, barrier function may be influenced by Cldn subcellular localization. In this context the observed changes in Cldn localization described in this study may be a result of multiple mechanisms including changes in Cldn phosphorylation. Although a number of Cldns, including the ones assessed in this study, have computer-predicted phosphorylation sites in the cytoplasmic tail domain, the functional significance of such sites has been demonstrated for only a few Cldns; e.g. Cldn3 where post-translational phosphorylation at threonine 192 has been shown to regulate TJ barrier function in ovarian cancer cells [16]. Another possibility is that there is increased endocytosis and/or an inhibition of a post-translational insertion into the ER resulting in Cldn retention in cytoplasmic vesicles. However, further investigation into the molecular mechanism(s) governing Cldn expression and localization in skin tumorigenesis is required.

### Changes in Cldn1 expression through epidermal tumor progression

**Figure 4:**
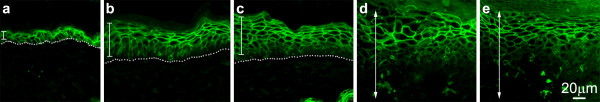
**Changes in Cldn1 expression in skin tumorigenesis**. In the normal (not shown) and vehicle-treated (**a**, after 12 weeks) epidermis, Cldn1 is localized in the basal and suprabasal layers; however in response to the two-stage chemical carcinogenesis protocol, the number of Cldn1-positive epithelial cells was progressively reduced starting from the basal layer and moving upwards at 2 (**b**), 4 (**c**), 8 (**d**) and 12 (**e**) weeks. Although a distinctly membranous Cldn1 association was maintained in the upper layers of the treated epidermis, as the number of Cldn1-negative epidermal cells in the lower epidermal layers increased, only sporadic Cldn1 localization was evident (**c-e**). The epidermal basal layer is indicated by a dotted line, and the suprabasal compartment is marked with a bracket (**a-c**); note that the basal layer is out of view in panels **d **and **e**; the entire view is therefore the suprabasal compartment and is marked with a double-ended arrow.

Amongst the Cldns in the epidermis, Cldn1 expression is unique and undergoes a maturation switch parallel to the acquisition of epidermal barrier function. In the developing epidermis, Cldn1 expression is first restricted to the stratified layers at E15.5, and by E17.5 it occupies both the basal and suprabasal compartments [4]. This mature expression pattern is maintained throughout life under normal conditions and was not modified when the epidermis was exposed to vehicle over the duration of tumorigenesis experiments (Figure 4a, the acetone-treated epidermis after 12 weeks is shown). However in response to DMBA/TPA treatment, Cldn1 lost its normal distribution pattern and adopted an expression like the immature pattern (Figure 4). Thus, 2 weeks after initiation of the carcinogenesis protocol, Cldn1-positive cells were decreased in the basal layer of the epidermis, while the entire suprabasal compartment maintained cell membrane-associated Cldn1 expression (Figure 4b); by 4 weeks of treatment, the basal layer was essentially devoid of Cldn1 (Figure 4c). After 8 weeks of treatment, the zone of Cldn1-negative cells expanded upwards into the lower strata of the suprabasal compartment, with only sporadic presence of Cldn1 protein remaining (Figure 4d), a phenomena more exaggerated after 12 weeks (Figure 4e). However, and in contrast to the normal suprabasal-specific Cldns, the membranous localization of Cldn1 was preserved throughout the 12 weeks of sampling in the upper strata of the suprabasal epidermis. Reminiscent of the reduced number of Cldn6, Cldn11, Cldn12 and Cldn18-positive epithelial cell layers in the characteristic epidermal structures infiltrating the dermis, these areas were completely devoid of Cldn1 expression (not shown).

Although the molecular mechanisms responsible for the reduced number of Cldn1-positive cells in epithelial tumorigenesis have not been defined, one possibility involves suppression by the Snail family of genes, which are involved in numerous tumors both in vivo and in vitro [17]. In support of this notion, functional Snail-binding E-box motifs have been demonstrated to have a suppressive role on Cldn1 expression in vitro [18,19]. Furthermore, a role for promoter methylation in the silencing of Cldn3, Cldn4 and Cldn7 has been observed in some tumors [20,21], suggesting that methylation could also play a role. Further studies are required to identify the mechanisms responsible for the changes observed in Cldn expression through epidermal tumor progression. However, our studies indicate that in this model the expression and distribution of Cldns change drastically and in a manner consistent with the loss of cell polarity and altered barrier selectivity concomitant with epidermal tumor formation.

## Abbreviations used

Cldns: Claudins;

TJs: Tight junctions;

DMBA: 7,12-dimethylbenz(*a*)anthracene;

TPA: 12-O-tetradecanoyl-phorbol-13-acetate.

## Competing interests

The author(s) declare that they have no competing interests.

## Authors' contributions

AA's contribution entails the acquisition and presentation of data. TT's contribution involves the generation of reagents, the presentation of data as well as the drafting and revising of the manuscript. KT has made substantial intellectual contributions to the conception, design, analysis and interpretation of the data in addition to revising the manuscript for intellectual content and has given final approval of the version to be published. All authors have read and approved the final manuscript.

## Pre-publication history

The pre-publication history for this paper can be accessed here:


